# Evaluating the neurophysiological evidence for predictive processing as a model of perception

**DOI:** 10.1111/nyas.14321

**Published:** 2020-03-08

**Authors:** Kevin S. Walsh, David P. McGovern, Andy Clark, Redmond G. O'Connell

**Affiliations:** ^1^ Trinity College Institute of Neuroscience and School of Psychology Trinity College Dublin Dublin Ireland; ^2^ School of Psychology Dublin City University Dublin Ireland; ^3^ Department of Philosophy University of Sussex Brighton UK; ^4^ Department of Informatics University of Sussex Brighton UK

**Keywords:** predictive processing, perception, neurophysiology, perceptual inference, predictive coding

## Abstract

For many years, the dominant theoretical framework guiding research into the neural origins of perceptual experience has been provided by hierarchical feedforward models, in which sensory inputs are passed through a series of increasingly complex feature detectors. However, the long‐standing orthodoxy of these accounts has recently been challenged by a radically different set of theories that contend that perception arises from a purely inferential process supported by two distinct classes of neurons: those that transmit predictions about sensory states and those that signal sensory information that deviates from those predictions. Although these predictive processing (PP) models have become increasingly influential in cognitive neuroscience, they are also criticized for lacking the empirical support to justify their status. This limited evidence base partly reflects the considerable methodological challenges that are presented when trying to test the unique predictions of these models. However, a confluence of technological and theoretical advances has prompted a recent surge in human and nonhuman neurophysiological research seeking to fill this empirical gap. Here, we will review this new research and evaluate the degree to which its findings support the key claims of PP.

## Graphical abstract

Predictive processing models have become increasingly influential in cognitive neuroscience as a possible explanation for the neural origins of perceptual experience, but have been criticized for lacking adequate empirical support. However, there has been a recent surge in human and nonhuman neurophysiological research seeking to fill this empirical gap. Here, we will review this new research and evaluate the degree to which its findings support the key claims of predictive processing.

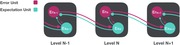

## Introduction

Our immersion in a seamless and coherent perceptual experience veils the reality that it must be assembled from a sea of noisy and ambiguous sensory information.^1^, ^2^, ^3^ It was Helmholtz who first proposed that perception does not obediently reflect the sensory inputs that undergird it, as exposed in myriad perceptual illusions,^4^, ^5^, ^6^ but arises from an inferential process in which stimuli are interpreted in light of our past experiences. Quite how this is achieved in the brain is still hotly debated.^7^, ^8^, ^9^, ^10^, ^11^ Thus far, the dominant framework guiding empirical research has been provided by feedforward models, which contend that our perceptual experience is assembled by a series of spatiotemporal filters that extract increasingly complex stimulus features as sensory information ascends through the cortical hierarchy.^12^, ^13^, ^14^ These traditional models account for the influence of prior knowledge on perception by invoking a variety of processing strategies and computational heuristics^15^, ^16^ (e.g., the inhibition of short‐range by long‐range motion signals to solve the correspondence problem) that are tailored for particular sensory features and contexts. The success of feedforward models in accommodating neurophysiological and anatomical observations has established them as the orthodox perspective on sensory processing. In recent years, however, a growing contingent of scholars has entertained a radical new framework that casts perception as an entirely inferential process in which predictions about the outside world shape information processing at all levels of the cortical hierarchy.^1^, ^4^, ^9^, ^17^, ^18^, ^19^

Predictive processing (PP) claims that the brain confronts the inherent ambiguity in sensory input by assembling “generative models” of the causes underlying sensory events. Generative models yield predictions about the pattern of sensory input that would be expected if the model's estimate of the cause was correct. According to the dominant neural process account of PP, known as predictive coding,^18^ these predictions are sent cascading down the processing hierarchy, suppressing congruent incoming sensory signals, such that only the residual, unexplained components of sensory information remain to be fed forward to higher levels in the form of “prediction error.” The brain's generative models continuously exploit these error signals to revise the probability assigned to perceptual hypotheses as this iterative process plays out across all levels of the processing hierarchy, until the network converges on a consistent representation of sensory causes. From this perspective, perception is the process of identifying the perceptual hypothesis that best predicts sensory input and hence, minimizes prediction error.^9^, ^20^

**Figure 1:**
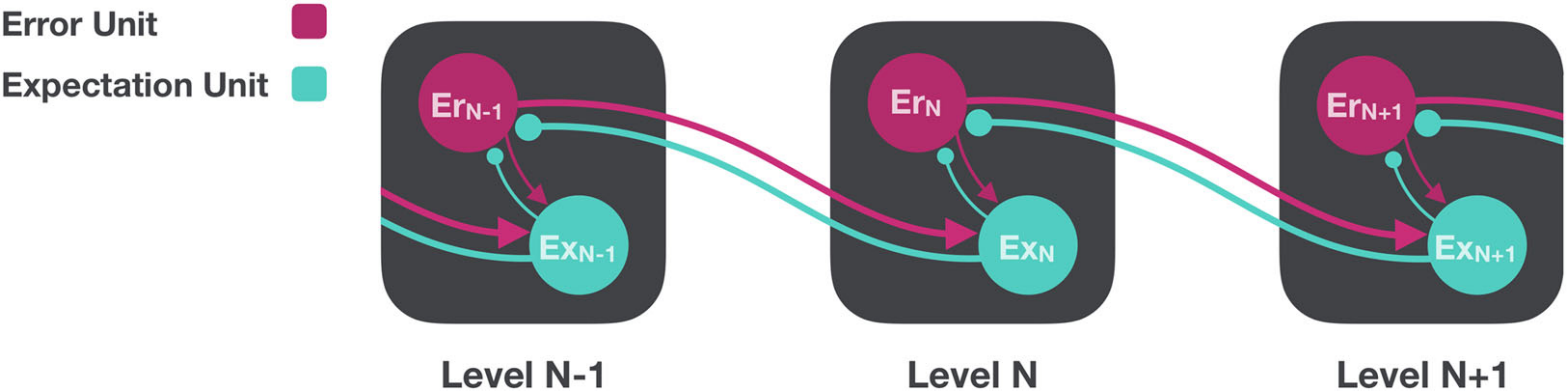
A simplified hierarchical PP model based on the circuit proposed by Rao and Ballard.^18^ Here, each level is composed of a population of reciprocally connected expectation units (Ex) and error units (Er). Expectation units signal the expected pattern of activity at the preceding level, given the current perceptual hypotheses. Where discrepancies emerge between these sensory expectations and sensory‐driven input, prediction errors are fed forward to the subsequent level to inform the revisions of prediction necessary to minimize prediction error.

PP claims that this process is neurophysiologically instantiated in an inferential hierarchy composed of two functionally distinct neural subpopulations: expectation units that communicate expected sensory states downward and laterally within the processing hierarchy, and error units that feed prediction error signals upward and laterally^9^ (Fig. 1). Importantly, prediction errors are not regarded as general surprise or arousal signals but rather, the source, connectivity, and stimulus preferences of an error unit imbue its output with specific information about the nature of the mismatch between predicted and actual input.^21^ Thus, where traditional models propose that forward signals emanating from primary visual cortex (V1) directly reflect stimulus orientation, PP proposes that they exclusively encode deviations from an expected orientation. Equivalently, where traditional models propose that inferotemporal cortex (IT) detects object identities, PP proposes that its error units signal only unpredicted object identities. In other words, PP hinges on an inversion of classical feedforward accounts whereby it is the descending signals that provide representations of the external world while forward signals provide the feedback that modifies those representations. Thus, PP has at its core a process of “predicting the present,” in which top‐down flows attempt to match incoming sensory stimulations, but they achieve this goal using information that spans many windows of space and timeaRecent work in the area also looks at even longer windows, selecting policies that aim to reduce expected future prediction error (see Ref. 22)..

To be effective, a PP system cannot treat all prediction error signals equally: sensory data can fail to conform to expectations because those expectations are incorrect or because the data are noisy or unreliable.^21^, ^23^ According to PP, the brain addresses this by adjusting the influence of particular sources of prediction error on perceptual hypotheses according to an estimate of the relative reliability associated with sensory evidence (i.e., sensory precision) and with higher‐level representations (i.e., prior precision).^9^, ^24^, ^25^ The “precision‐weighting” assigned to prediction error is encoded in the synaptic gain applied to the associated error units, such that high‐precision errors exert greater influence in commanding revisions of perceptual hypotheses, while current predictions are more stubborn in the face of low‐precision errors.^26^ Hence, precision‐weighting provides a mechanism through which the brain can promote the influence of error channels that will provide the most valuable information in revising sensory expectations (see “Precision and attention” section).

A compelling feature of PP models is that they specify a single mechanism that naturally accounts for a range of prominent perceptual and neural phenomena ranging from end‐stopping^18^ to repetition suppression (RS) and error responses,^27^ attentional modulations of sensory signals,^28^ bistable perception,^29^ and motion illusions.^30^, ^31^ But perhaps the most seductive aspect of these models is their apparent potential to provide a unified theory of the mind, with several theorists suggesting that hierarchical inference is the fundamental mechanism underlying all neural computation^1^, ^9^, ^17^, ^19^, ^21^, ^32^ (see Ref. 23 for a critical review). However, an oft‐repeated criticism of PP is that it simply lacks the empirical foundation to undergird these grand claims^10^, ^33^, ^34^, ^35^, ^36^ (see commentaries on Ref. 17). In reality, many of the perceptual and neurophysiological phenomena that are regularly highlighted as evidence in favor of PP can also be accommodated within traditional models by invoking additional mechanisms (e.g., neural adaptation in the case of RS). Most of PP's unique predictions relate to fine‐grained neuronal and circuit‐level phenomena that require carefully tailored behavioral paradigms and sensitive neural assays. A variety of PP‐consistent modelsbNote that there are other biologically plausible accounts of predictive processing (e.g., belief propagation and variational message passing), which many take to be synonymous with active inference, although they would not necessarily involve prediction error minimization, but here we focus on predictive coding because it has been by far the dominant account considered by the extant neurophysiological literature. have been proposed,^2^, ^9^, ^18^, ^37^, ^38^, ^39^, ^40^, ^41^ but despite this heterogeneity, there are a number of shared, canonical features that clearly dissociate PP from traditional models of perception. To date, neurophysiological investigations of PP have largely centered around testing four key hypotheses:

1. Error‐signaling neural responses to sensory stimuli should scale inversely with expectation.
2. Top‐down signals represent sensory prediction.
3. At each level of the cortical hierarchy there are two functionally distinct neural subpopulations representing predictions and prediction errors.
4. Prediction error minimization is achieved through reciprocal exchange of error and prediction signals across levels—a process known as “hierarchical inference.”

**Figure 2:**
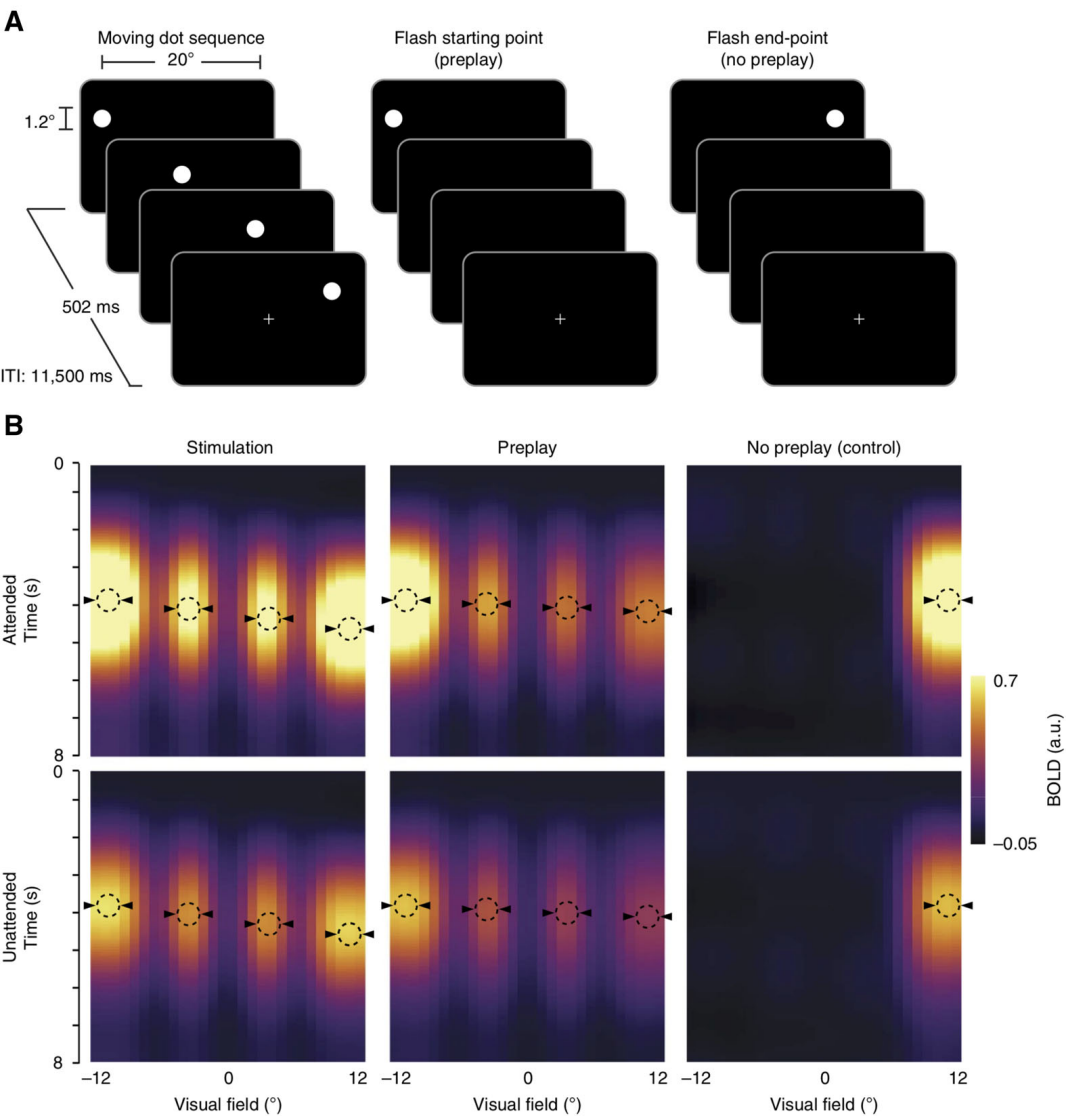
Task schematic and data from Ekman *et al*. (adapted from Ref. 51). (A) Human participants were repeatedly presented with a moving dot sequence for 4 minutes. Following this dot sequence, participants were then shown either the starting point of the sequence or the endpoint, which was briefly flashed on the screen. (B) The starting‐point stimulus generated a sequence of BOLD activity across the retinotopic locations of V1 corresponding to the positions of the actual dot stimulus, which reconstructed the stimulus sequence in a time‐compressed format (i.e., this activity unfolded more rapidly than the response to the actual stimuli). This “preplay” of the stimulus sequence was not elicited by the endpoint stimulus and was still observed in the absence of attention. The authors argued that the time‐compressed format indicated that this represented automatic predictive activity and not surprise at the omitted stimuli. Enhanced activity in hMT indicated that this activity is fed back from higher‐level regions.

**Figure 3:**
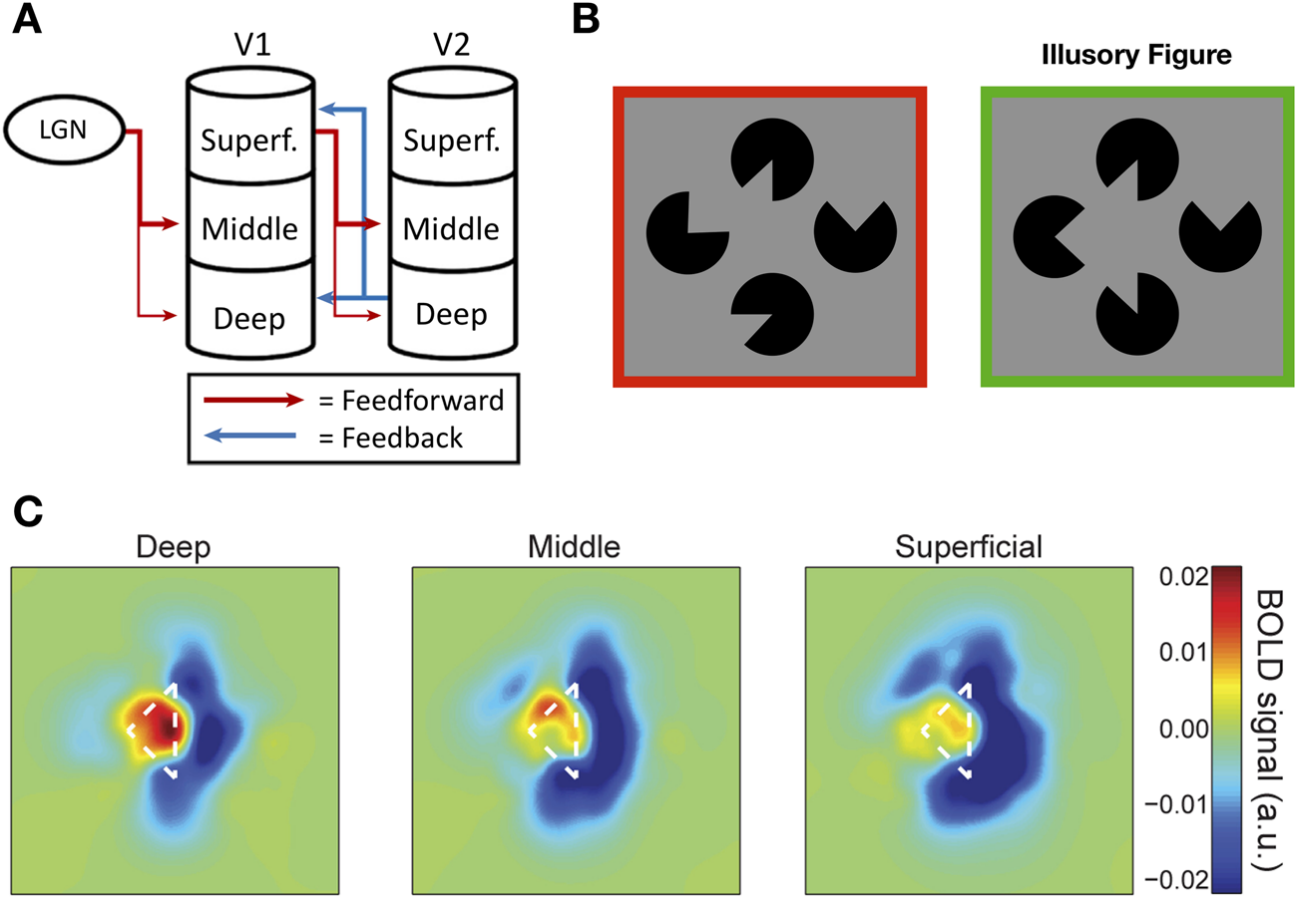
(A) The laminar segregation of feedforward and feedback connections between human lateral geniculate nucleus, V1, and V2. According to PP, expectation units are expected to primarily occupy deep cortical layers, while error units should be found in superficial layers (adapted from Ref. 21^4^). (B) Kok *et al*.^169^ presented participants with a Kanizsa illusion while recording their BOLD response with 7T fMRI. The task is well poised to isolate prediction signals because the bottom‐up sensory input is the same whether the illusory triangle is presented or not. (C) Data from Kok *et al*. demonstrating the pattern of BOLD activity across different cortical layers in response to a Kanizsa triangle illusion and its “Pac‐Man” inducers. The results showed significantly enhanced activity in deep layers of V1 corresponding to the illusory triangle, which could be interpreted as representing the activity of expectation units signaling the presence of the illusory triangle. There was also evidence of suppressed activity in middle and superficial layers of V1 where the Pac‐Man inducers fell within the receptive fields. This response suppression exhibited the same laminar profile as the response to a checkerboard stimulus (adapted from Ref. 169).

**Figure 4:**
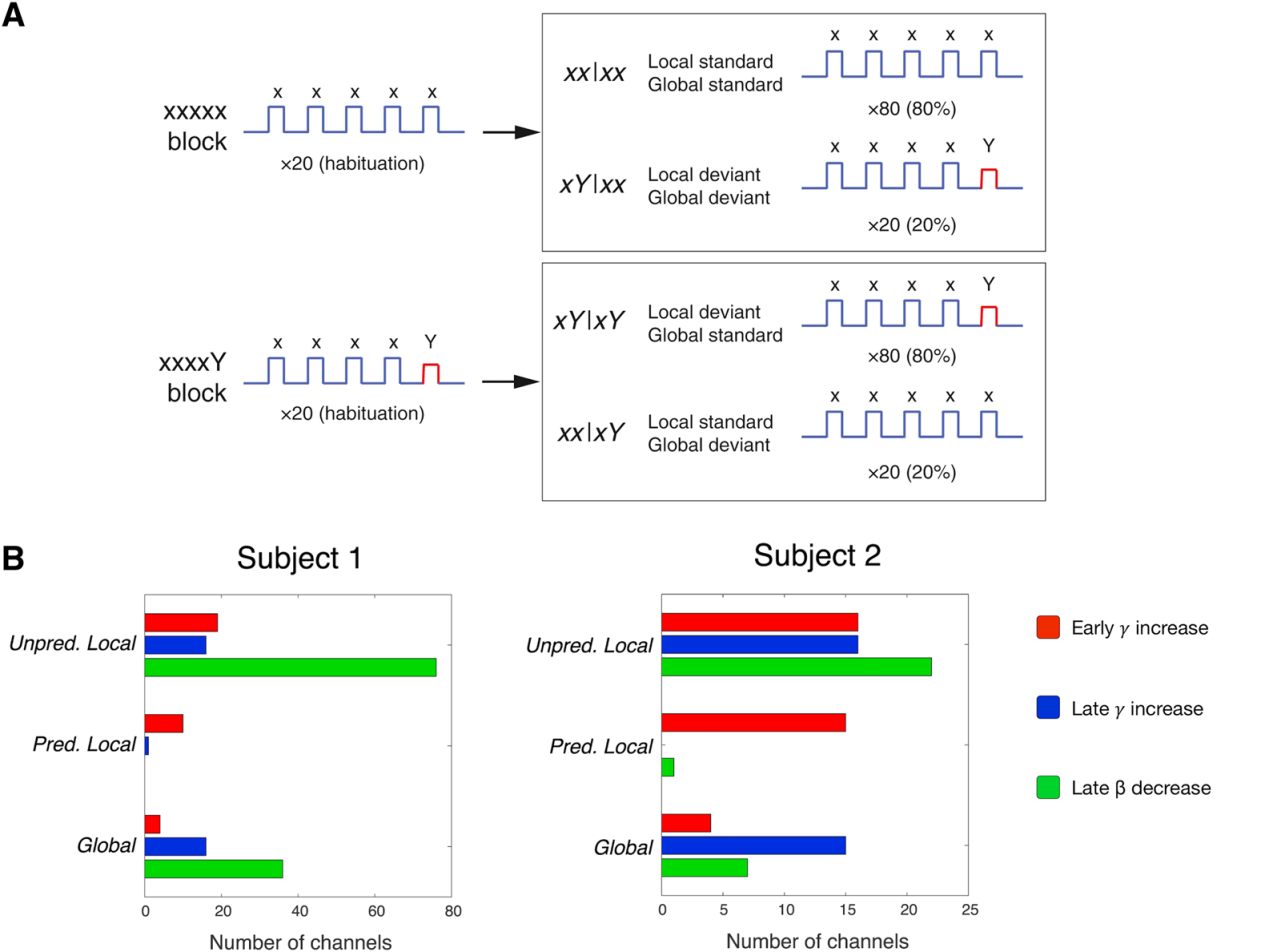
Experimental paradigm and resulting data from Chao *et al*. (adapted from Ref. 158). (A) Chao *et al*. used an auditory local–global paradigm, whereby local and global regularities could be respected or violated, to investigate the dynamics of oscillatory activity in macaque monkeys using ECoG recordings. Let us assume the standard (expected) sequence is xxxxY. If the sequence presented to the subject is xxxxY, “Y” represents a local deviant (differing from the preceding tone, but not the global pattern). If the sequence played was xxxxx instead, the final “x” represents a global deviant (differing from the global pattern, but not the local pattern). (B) The results showed three components that closely matched model estimates for a low‐level prediction error, a higher‐level prediction error, and a prediction update signal. The low‐level prediction error was associated with an early gamma‐power increase over primary auditory cortex and was elicited by unpredicted local deviants. The higher‐level prediction error was composed of a late phase gamma‐power increase over anterior temporal cortex and was elicited by local and global deviants. Finally, the prediction update component was represented by a late beta‐power decrease over prefrontal cortex, which followed the higher‐level prediction error, and was associated with unpredicted local and global deviants. The authors found that when both local and global regularities were violated, the activation of the prediction update signal significantly affected activation levels of the low‐level prediction error and higher‐level prediction error on the subsequent trial. When only the global regularity was violated, the prediction update component's activation only influenced the higher‐level prediction error on the next trial.

**Figure 5:**
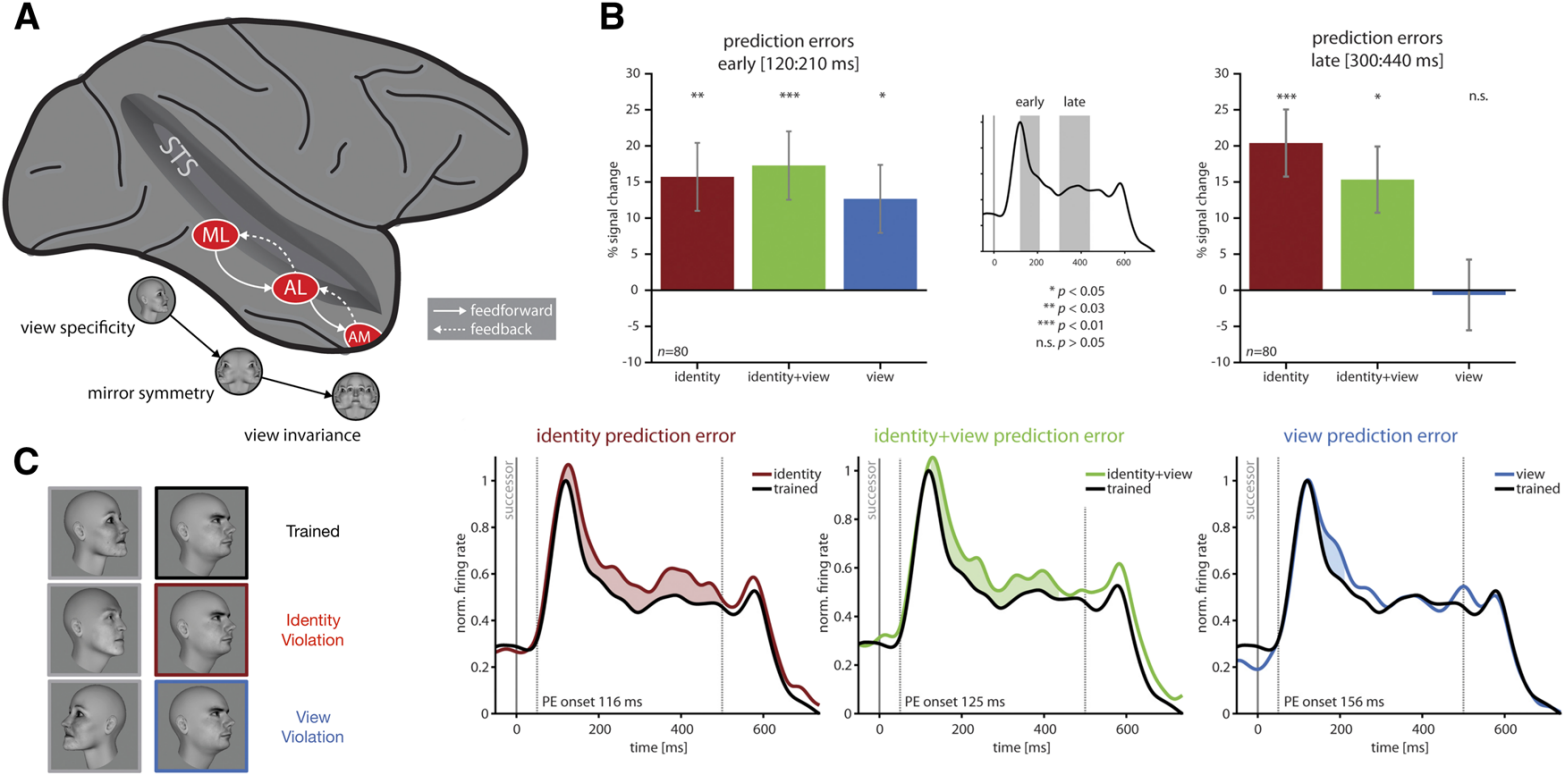
The macaque face processing system in IT and prediction error responses elicited from its constituent cortical areas (this figure was adapted from Ref. 92). (A) The macaque face processing network is composed of three face‐selective areas, each with its own functional specialization, arranged in a three‐level processing hierarchy. Although tuning to head orientation decreases from area ML via area AL to area AM, selectivity for facial identity across head orientations increases.^73^ (B) Schwiedrzik and Freiwald^92^ recorded single‐unit activity in ML in response to learned face pairs, where occasional deviant stimuli had a different identity, head position, or both. Although all three violations individually produced a significant early prediction error response, the late sustained response to orientation violations dwindled and disappeared, but the response to identity and combined violations remained significant. These findings suggest that early responses reflect mismatches along local tuning properties, but later responses reflect view‐invariant identity errors. Mirror‐symmetric view violations evoked smaller prediction errors in ML than nonmirror‐symmetric violations in the late phase. As the timing of this effect overlaps with the peak of mirror‐symmetric identity tuning in AL, this suggests that higher order representations are involved in suppressing prediction errors in the late phase. (C) However, the interpretation of these results is complicated by the coding scheme where the deviant stimuli were successors to untrained predictors rather than unexpected successors to trained predictors.

**Figure 6:**
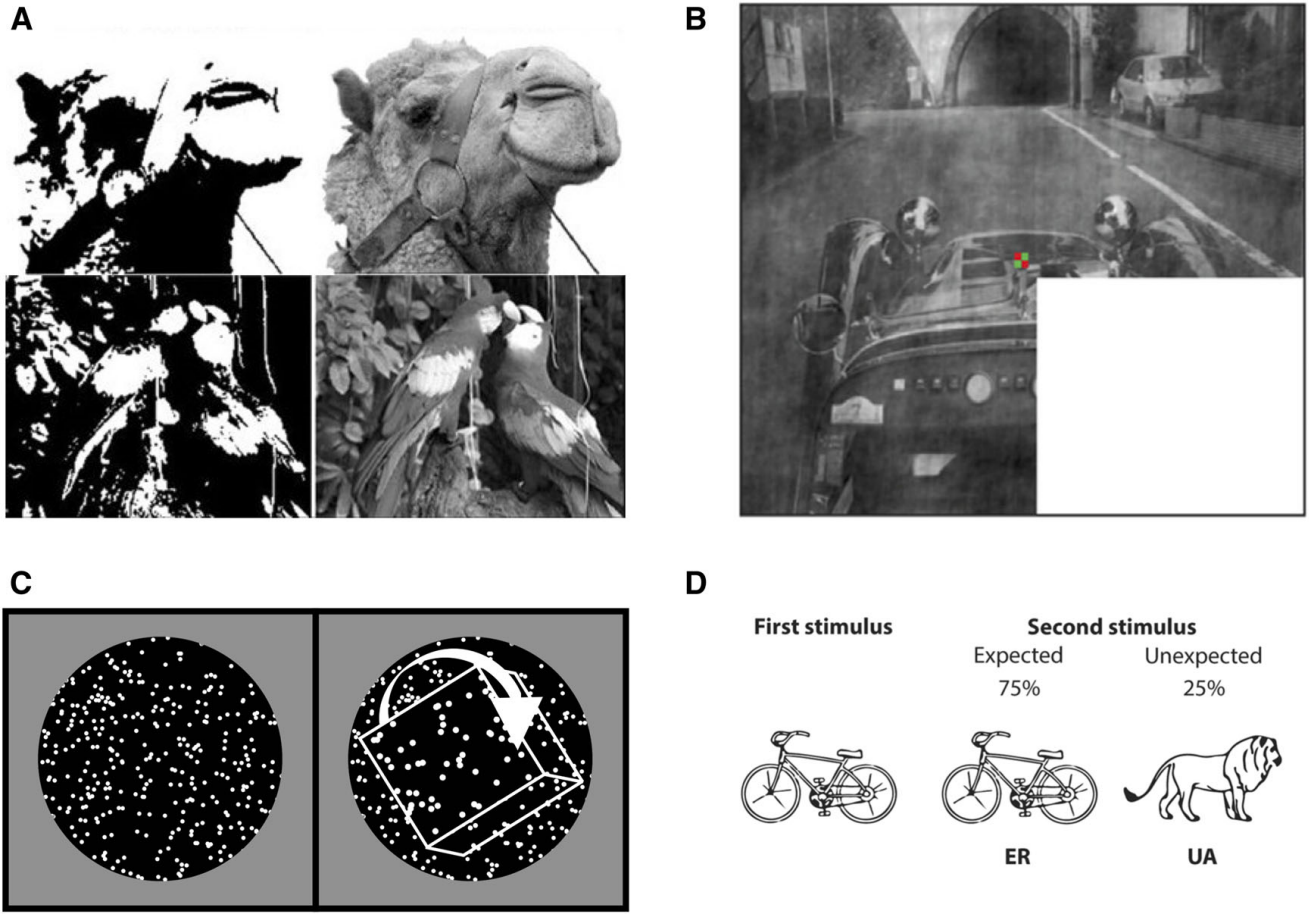
Examples of stimuli used to probe the nature of sensory prediction. (A) Mooney images used by Hsieh *et al*. (adapted from Ref. 199). The two tone image becomes interpretable when the full greyscale image is seen. (B) An occluded visual scene used by Muckli *et al*. (adapted from Ref. 132). Their analyses were conducted on V1 neurons whose receptive field fell within the occluded region. (C) An illustration of the structure‐from‐motion stimulus used by Murray *et al*.^115^ (D) Expected and unexpected stimulus pairs used by Utzerath *et al*. (adapted from Ref. 67).

In the last 5 years, methodological advances and the increasing reach and influence of PP in the neurosciences have prompted a significant surge in the number of neurophysiological investigations seeking to definitively test these claims. Here, we seek to offer a balanced overview and critical analysis of the current state of the evidence, highlighting some of the key studies that exemplify recent progress in the field (see Figs. 2, 3, 4, 5, 6).

## Hypothesis 1: Error‐signaling neural responses to sensory stimuli should scale inversely with expectation

The vast majority of neurophysiological investigations of PP have centered on the proposal that error‐signaling neural responses to sensory stimuli should scale inversely with expectation. Much of what has been considered the core evidence for PP comes from experiments that test for three basic phenomena: vigorous neural responses to the omission of an expected stimulus (the “omission response”), the suppression of neural activity following repeated stimulus presentations (“repetition suppression”), and the suppression of neural activity following stimuli that are expected on the basis of statistical regularities or prior cues (“expectation suppression”). As we will see in this section, research in this domain has relied heavily on noninvasive recording methods that provide population‐level measures of brain activity. This is potentially problematic because, in many instances, PP makes opposite predictions regarding the impact of a given experimental manipulation on expectation versus error unit activity and it is not clear how these distinct modulations should manifest in global neural responses. Although this is not a reason to dismiss the neural phenomena reported in this section, it is an important caveat when relating them to PP.

### Omission responses

Perhaps the most compelling example of the putative prediction error signal is the robust neural response elicited by the *omission* of an expected stimulus^42^, ^43^, ^44^, ^45^, ^46^, ^47^, ^48^ (see Ref. 42 for a review). Importantly, rather than being generic surprise responses, several studies have identified omission signals that are feature‐selective. For example, omission responses in primary visual cortex have been shown to be retinotopically specific and exhibit comparable feature specificity with the responses that are evoked when the stimulus is presented.^49^ Although PP can comfortably accommodate prediction error signals being elicited in the absence of any sensory input, such omission responses do not appear easy to reconcile with feedforward models that are devoid of any predictive influence on sensory processing^41^ (but see Ref. 50). However, PP's interpretation of the omission response also suffers from some ambiguities. For example, if prediction is simply subtracted from bottom‐up activity, the prediction error response to the omission would require negative firing rates (see “Evidence of prediction in sensory processing” section). This has led some to suggest that omission responses may predominately reflect the activity of expectation units rather than error units.^37^ Indeed, it might be hypothesized that the omission response is composed of activity in expectation units representing the sensory prediction of the absent stimulus^51^, ^52^, ^53^ and the error response to the mismatch with those expectations. Thus, while the observation of neural responses to unexpected stimulus omissions provides evidence for the role of prediction in sensory processing, further research is required to work out their precise neurophysiological origins.

### Repetition suppression

RS has been consistently reported across a wide range of methodologies, sensory modalities, stimulus properties, and time scales.^54^, ^55^ Although it accords neatly with PP,^27^ an equally plausible interpretation of RS is that it arises from low‐level changes in the responsivity of stimulus‐selective neurons (i.e., neural adaptation).^56^ Thus, the key test for PP models is not whether RS occurs, but whether its occurrence can be directly attributed to expectation. An influential early study by Summerfield *et al*.^57^ sought to control for the effects of adaptation by comparing blood oxygen level—dependent (BOLD) activity in the fusiform face area (FFA) of human participants in response to stimulus repetitions that were expected versus unexpected, and found that expected repetitions yielded substantially smaller BOLD responses. Numerous subsequent functional magnetic resonance imaging (fMRI), magnetoencephalography (MEG), and electroencephalography (EEG) studies have reported modulations of RS by the probability of a repetition^45^, ^46^, ^58^, ^59^, ^60^, ^61^, ^62^, ^63^, ^64^, ^65^, ^66^, ^67^ (i.e., expectation) (but see Refs. 68, 69, 70). Also consistent with PP, and seemingly at odds with neural adaptation accounts, is evidence that unexpected repetitions evoke greater neural responses than frequent alternations.^37^ However, as we will discuss in more detail below (Hypothesis 4), PP asserts that expectations due to stimulus repetition and those due to knowledge of stimulus probability emanate from distinct processing levels. Grotheer and Kovacs^61^ implemented orthogonal manipulations of face repetition and repetition probability, noting that studies potentially confound the effects of repetition and expectation on sensory signals by manipulating the relative probability of repetitions and alternations across blocks. They observed independent, additive effects of each in FFA, the occipital face area, and the lateral occipital complex (LOC).

Attempting to replicate the findings of Summerfield *et al*.^57^ with single‐cell recordings in macaque IT, Kaliukhovich and Vogels^71^ reported that responses to deviant stimuli were indistinguishable from responses to infrequent, but conditionally probable, stimuli. However, it is unclear to what extent this discrepant result reflects certain methodological differences. First, Kaliukhovich and Vogels used stimuli that were unfamiliar to the monkeys, but some human studies indicate that RS expectation effects are most pronounced for stimuli that are highly familiar to the observer^59^, ^61^ (e.g., letters or faces; but see Ref. 63). Second, the task demanded only passive fixation rather than attentional engagement, which has been shown to strongly affect the modulation of RS by expectation.^60^ Third, Kaliukhovich and Vogels recorded from IT, while Summerfield *et al*. measured activity in FFA. To address these issues, Vinken *et al*.^72^ recorded neural activity in response to face stimuli in the middle lateral face patch (ML), a macaque homolog of FFA. Once again, they failed to observe any evidence of an effect of repetition probability on RS in single‐unit or multiunit activity, even when the repetitions were task‐relevant and repetition probability was found to affect the monkeys' behavior. However, Vinken *et al*. measured responses from ML, which is characterized as having view specificity rather than identity specificity.^73^ As alternate stimuli had a different identity, but a similar viewpoint, these stimuli may not have optimally differentiated “expected” and “unexpected” responses. Importantly, the results from both Vinken *et al*. and Kaliukhovich and Vogels were presented as single population analyses, which would also obscure any functional heterogeneity among individual neurons representing error units or expectation units. Thus, it remains an open question if the effects of RS are a consequence of perceptual expectations,^27^, ^55^ but the fact that PP can accommodate RS as a purely low‐level phenomenon^27^ suggests that RS may not represent a useful vehicle for definitively adjudicating between PP‐ and feedforward/adaptation‐based accounts.

### Expectation suppression

A substantial body of work has also sought to test PP predictions pertaining to expectation suppression (ES) using a variety of paradigms (e.g., predictive cues, paired associations, and predictable stimulus sequences). As with RS, ES has been consistently reported in human brain imaging studies across sensory modalities and noninvasive neural recording techniques^45^, ^46^, ^74^, ^75^, ^76^, ^77^, ^78^, ^79^, ^80^, ^81^ (but see Ref. 82) and a number of human studies have demonstrated that the magnitude of stimulus‐evoked responses is inversely proportional to the degree to which the stimulus was expected^75^, ^76^, ^83^, ^84^ (see Refs. 47 and 85 for corresponding evidence from neuronal recordings in rodents and monkeys, respectively). A key observation from this work has been that the observed neural modulations are not generic to broad categories of stimuli, but specific to individually predicted exemplars. For instance, Pajani *et al*.^70^ observed ES in FFA BOLD activity in response to specifically predicted facial identities relative to nonpredicted faces.

Although many of the above studies could not segregate the distinct contributions of error and expectation units to the observed global neural response modulations, some studies have applied alternative experimental designs and methodologies that were tailored to do so. Egner *et al*.^76^ examined BOLD responses to face and house stimuli that were either expected or unexpected, and observed effects consistent with ES in FFA. The authors also made the surprising observation that, despite its well‐established role in face processing,^86^ FFA responses to faces and houses were statistically indistinguishable when those stimuli were expected. The full pattern of effects was best explained by a PP model in which BOLD responses were dominated by strong prediction‐driven, face‐selective expectation unit activity and weak face‐selective error unit activity on both trial types (since houses would evoke minimal error signals from face‐selective cells). Another fMRI investigation by de Gardelle *et al*.^87^ attempted to isolate activity attributable to each subpopulation when examining RS. The authors hypothesized that activity in expectation units should be enhanced as repetitions generate increasingly precise sensory expectations, while error units should exhibit RS as these predictions eradicate prediction error. Consistent with these predictions, two segregated clusters of FFA voxels were found to exhibit either RS or repetition enhancement. Although the authors demonstrated that the classification of each voxel was stable across measurements, the inevitable intermingling of the proposed expectation and error unit populations within each 3‐mm cubic voxel is a limitation of this approach. For example, Auksztulewicz and Friston^27^ point out that increased expectation‐linked activation of a given voxel cannot be reliably attributed to expectation unit activity because the same trend could arise if prediction error units in that voxel had been assigned increased precision‐weighting as the task statistics are learned.

A series of single‐ and multiunit recording studies have also reported evidence of ES in macaque IT neurons.^85^, ^88^, ^89^, ^90^, ^91^, ^92^ These effects were sensitive to transitional statistics (e.g., the transition from Stimulus A to Stimulus B being more probable than from B to A),^90^, ^91^ persisted after controlling for RS,^88^, ^89^ and emerged at early latencies (∼150 ms).^88^, ^90^, ^91^, ^92^ For example, Bell *et al*.^88^ recorded single‐unit activity from IT neurons while monkeys performed a delayed match‐to‐sample task in which they indicated which of two stimuli (a face or a fruit) best matched a previously displayed cue stimulus that was degraded by noise. They measured the effect of stimulus probability on IT responses by manipulating the relative probability of the cue being a face or a fruit, which changed unpredictably over the course of the recording session. The results showed a reduced population response to expected faces in face‐selective IT neurons, which Bell *et al*. interpreted as evidence that IT neurons encode prediction errors and long‐term probabilistic information, in line with PP. Although many of these invasive ES studies are subject to the same criticism of failing to segregate expectation unit and error unit activity as the studies from the RS literature, the analyses of Bell *et al*.^88^ did identify two distinct populations underlying the recorded activity (see Hypothesis 3), with the ES effect evident in the putative error unit subpopulation specifically.

However, Vinken and Vogels^93^ showed that this reduced response in IT neurons could be reproduced in a simulated population of neurons through stimulus‐specific neural adaptation without recourse to PP mechanisms. The authors simulated a population of neurons displaying stimulus‐selective responses that decreased with each presentation of a preferred stimulus and recovered between presentations, allowing suppression to build up over time. This build‐up of suppression over time ultimately accounted for the putative ES effects, indicating that they could be explained by passive adaptation mechanisms;^93^ although there has been subsequent debate as to whether the long time window over which adaptation operates in the simulations is realistic given previous empirical observations.^94^ The picture is further complicated by the fact that a number of direct recording studies have produced the opposite pattern of results to *Bell et al*., with unexpected or random stimuli eliciting reduced responses relative to expected or neutral stimuli in macaque IT^95^, ^96^ and rodent V1^52^ (see Ref. 97 for corresponding results in a human study). There is also conflicting evidence regarding whether IT neurons are sensitive to statistical regularities present in nonadjacent stimuli from a sequence of images^91^ or whether these suppression effects are determined by the immediately preceding stimulus.^89^, ^96^

### Precision and attention

One promising avenue for disentangling PP and adaptation‐based accounts arises from PP's specification that the magnitude of expectation effects on neural activity depends on the precision‐weighting applied to particular error signals. To efficiently minimize prediction error, the system adjusts the precision‐weighting assigned to prediction error according to the estimated reliability of sensory information. Hence, the amplitude of error responses will reflect both differences in top‐down predictions and differences in predicted precision. Indeed, as mentioned above, many studies have reported that stimulus‐evoked responses are inversely proportional to the degree to which the stimulus was expected,^47^, ^75^, ^76^, ^83^, ^84^, ^85^, ^98^ although this effect is not universally observed.^79^, ^82^

In PP, stimulus salience and attention are considered emergent properties of this precision‐weighting mechanism.^9^, ^28^ Here, attending to a stimulus feature or to a part of the visual field is equivalent to predicting high‐precision information from the associated error units and hence upweighting their error signals’ influence in subsequent revisions of perceptual hypotheses. This is consistent with the results of numerous studies that report larger neural responses to attended compared with unattended stimuli.^99^, ^100^, ^101^ Although this finding is easily reconciled with the traditional feedforward account of attention, PP diverges from this account in proposing an interaction between attention and expectation. Characterizing the nature of this interaction has proven difficult because the experimental designs found in much of the earlier research confounded attention and expectation^3^ and the heterogeneity of results from subsequent efforts to independently manipulate these variables precludes any conclusive resolution. For example, the prominent interaction model suggests that the error response to expected stimuli should be amplified for attended, but suppressed for unattended, stimuli, while the reverse should be true for unexpected stimuli.^102^, ^103^ Although several reports are consistent with this pattern,^102^, ^104^, ^105^ others have found that ES is abolished in the absence of attention,^60^, ^106^, ^107^ or that attention provides an equivalent boost to predicted and unpredicted stimuli.^77^ Hsu *et al*.^108^ argue that the source of these conflicting results may be the distinction between unpredicted and mispredicted. They found that the response suppression evident in comparisons of predicted and mispredicted stimuli showed no modulation by attention. However, when comparing responses with predicted and unpredicted stimuli, attention reversed ES.

### Hypothesis 1: Summary and conclusions

Although many studies report the expectation‐related neural modulations that are predicted by PP across the full range of neurophysiological recording techniques, a significant contingent failed to replicate these effects. For instance, there is a clear disparity in the frequency with which expectation‐related modulations are detected using global measures compared with direct neuronal recordings. The uncertainty surrounding the relationship between the neural activity preferentially recorded with invasive versus noninvasive assays^54^ also hinders efforts to integrate observations across the literature. In addition, this summary of the recent literature highlights considerable variation in the paradigms that have been employed. For instance, while some studies instill expectations by allowing subjects to learn stimulus probabilities through exposure (e.g., Ref. 66), others train subjects for days (e.g., Ref. 92); some manipulate baseline probabilities (e.g., Ref. 63), others manipulate transitional probabilities (e.g., Ref. 90); some engage attention (e.g., Ref. 45), others involve passive viewing (e.g., Ref. 71); some inform subjects about the manipulation of conditional probabilities (e.g., Ref. 81), others do not (e.g., Ref. 57). This methodological heterogeneity precludes direct comparisons across studies since PP makes divergent predictions regarding the properties of prediction error given different permutations of these variables.

Another source of uncertainty in interpreting these effects arises from the fact that there is no consensus about precisely which components of neural activity are being suppressed by expectation (see Ref. 4 for a recent review). Although some studies have reported that expected stimulus representations are dampened,^69^, ^79^, ^90^, ^92^, ^105^, ^109^, ^110^, ^111^ others have found that this global suppression is attributable to the suppression of neurons tuned away from the expected stimulus feature, while the representation of the expected stimulus is actually sharpenedcWhen interpreting these results, it should be noted that these decoding analyses of global BOLD activity or single‐unit responses did not segregate activity from the proposed error unit and expectation unit populations..^78^, ^88^, ^112^ Interestingly, Marques *et al*.^113^ recently found that while backward inputs from rodent lateromedial visual area (LM) to V1 were retinotopically matched on average, they also divaricated widely beyond the target receptive field, relaying distal visual information. In fact, half of V1 inputs from LM had receptive fields displaced more than 24° from their target in V1 and these axons increasingly targeted cells with the opposite tuning profile as the retinotopic distance between the LM and V1 cells increased, betraying a complex circuitry that would be difficult to uncover using global measures of brain activity. Additionally, as ES is typically calculated by comparing expected and unexpected stimulus responses, it is often unclear if it represents genuine suppression, suppression relative to the enhanced response to deviant stimuli, or both.^55^, ^88^, ^96^ For example, Kaposvari *et al*.^89^ provided evidence of both: an early transient suppression of the neural response to expected stimuli and later sustained enhanced activity in response to unexpected stimuli, reminiscent of a prediction error response.

A central criticism of many of the studies addressing Hypothesis 1 is the tendency to attribute fluctuations in feature‐selective global neural responses to error signals while overlooking the fact that, according to PP, expectation units are concurrently active and undergo distinct modulationsdIt should be noted that some have suggested that EEG recordings will preferentially sample the activity of error units due to their association with superficial pyramidal cells (e.g., Refs. 9 and 24; see Hypothesis 3)..^34^ Within a PP framework, global measures of neural activity, such as MEG/EEG and fMRI, will necessarily reflect a summation of activity from each subpopulation and the magnitudes of their relative contributions are not easily derived *a priori*. This is particularly important because PP makes opposite predictions depending on whether activity represents the transmission of sensory predictions^49^, ^82^, ^97^, ^114^, ^115^ (enhanced activity) or the integration of these predictions with bottom‐up activity in prediction error units (reduced activity).^45^, ^46^, ^74^, ^75^, ^76^, ^77^, ^78^, ^79^, ^80^, ^81^ Although this problem is not unique to PP, the indefinite profile of neural activity captured in BOLD activity^116^, ^117^ and MEG/EEG signals^118^ hampers efforts to provide dispositive evidence of PP in humans.^34^

However, PP does make a number of more detailed specifications regarding the architecture of the inferential circuitry that recent work has begun to address. First, PP proposes that predictive signals carried in descending cortical connections are responsible for the suppression of prediction error responses to expected stimuli (Hypothesis 2). Second, PP suggests that these signals originate in two functionally distinct neural subpopulations (error units and expectation units) and that their activity should reflect expectations in very different ways (Hypothesis 3). Third, PP proposes an inferential hierarchy whereby expectation modulations are not uniform across the cortex but are selectively applied at the processing levels to which the expectations pertain (Hypothesis 4). Thus, the particular neural populations and processing levels that are preferentially sampled by different recording techniques may be key to the study outcome. This may account for the fact that, in several instances, RS/ES effects that were observed with one noninvasive recording method were absent in another despite using otherwise identical paradigms.^53^, ^78^, ^119^ This issue greatly complicates efforts to generate precise, empirically testable PP hypotheses and also reduces the scope for disconfirmatory results.^42^ In recognition of this point, researchers are increasingly seeking to verify the other hypotheses of PP by attempting to isolate mechanisms underlying the generation of predictions and hierarchical inference, and it is to this literature that we now turn our attention.

## Hypothesis 2: Top‐down signals represent sensory predictions

A defining characteristic of PP is a cascade of descending predictions distributed to each level of the processing hierarchy to quell emerging prediction error.^17^ Human and animal studies indicate that the brain is finely tuned to efficiently extract regularities from streams of sensory input^120^, ^121^ through passive sensory experience,^52^, ^122^ self‐generated actions,^123^, ^124^ and exploration of the environment,^47^, ^124^ and that these expectations influence sensory processing.^45^, ^74^, ^78^, ^125^, ^126^ Although many agree that feedback plays an essential role in myriad sensory processes,^127^, ^128^ the neural instantiation of these influences is more controversial. As outlined in the preceding section, the vast majority of studies examining PP have tended to focus on prediction error responses, but a number of recent studies have also sought to probe the nature of neural prediction itself.

### Evidence of prediction in sensory processing

Devising experimental paradigms that can isolate stimulus‐specific prediction signals from other coincident neural activity is far from straightforward. One creative approach has been to exploit the Kanisza illusion in which “Pac‐Man” shapes are rotated to manipulate the perception of an illusory triangle (Fig. 3B) in order to probe for prediction responses in the absence of any bottom‐up input. In a departure from much of the work described above, these predictions emerge from the interpretation of a currently viewed stimulus rather than being based on experimentally controlled stimulus probabilities. Employing the Kanisza paradigm, Kok and de Lange^129^ observed increased BOLD activity in regions of V1 and V2 retinotopically mapped to the illusory triangle contours. However, it is not clear whether this activity should be interpreted as representing error unit activity arising from the absence of sensory input where triangle contours are expected or prediction unit activity reflecting the perceptual hypothesis itself.^34^ Single‐unit recording studies have indicated that neural responses to illusory contours reflect the descending influence of higher‐level areas.^130^, ^131^ For example, while both macaque V1 and V2 neurons respond to the illusory contour of a Kanizsa triangle, V2 neurons consistently respond earlier than those in V1.^130^ Several fMRI studies have also reported that patterns of neural activity evoked in early visual cortex following the omission of an expected stimulus resemble the activity evoked by the veridical stimulus^49^, ^51^, ^132^ and this result has been replicated in recordings from rodent V1.^52^ Muckli *et al*.^132^ occluded a subregion of a visual scene and isolated neural activity in voxels retinotopically mapped to this area of the visual field (Fig. 6B). In line with the idea that predictive signals descend from higher‐level processing areas, they observed that the classification performance of their decoding analysis was robust to 2° shifts in the visual scene, suggesting that this backward activity originates in neurons with larger receptive fields.

Elsewhere, several studies have offered evidence that sensory prediction may also rely on representations of stimulus trajectories leading into the future. For example, Bendixen *et al*.^43^ played participants a series of pairs of identical tones and, in certain instances, omitted either the first or second tone in a pair. Omission responses were only observed when the identity of an omitted tone was known in advance of its omission (i.e., the second tone of the pair was omitted; see also Ref. 44). Also consistent with this aspect of PP, enhanced anticipatory feature‐selective activity preceding high‐probability stimuli compared with low‐probability stimuli has been reported in MEG recordings of visual cortex,^53^ BOLD activity in FFA,^82^ and single‐unit recordings in macaque IT.^88^ Bell *et al*.^88^ and Kok *et al*.^53^ showed that a representation of the expected stimulus could be decoded from neural activity before stimulus onset using multivariate analyses and forward‐modeling, respectively. However, it should be noted that the prestimulus expectation effects observed by Trapp *et al*.^82^ and Kok *et al*.^53^ did not influence the subsequent stimulus‐evoked response.

In another line of research, Van Kerkoerle *et al*.^133^ trained monkeys to mentally retrace a briefly presented (150 ms) curve and recorded V1 activity. Following the disappearance of the curve stimulus, spiking activity persisted in superficial and deep layers of V1 in the same retinotopic locations as the presented contour, suggesting that V1 contains a persistent trace of recently presented stimuli. Interestingly, the activity was present up to 600 ms after stimulus offset and was temporarily erased by the presence of a visual mask, but reinstated on mask removal, demonstrating that the activity could not be explained through iconic memory mechanisms.^133^ In a human fMRI study, Ekman *et al*.^51^ repeatedly exposed participants to a dot stimulus rapidly moving across a screen. Following the exposure period, a flash of the dot stimulus at the starting position produced a time‐compressed sequence of BOLD activity across the retinotopic locations of V1 corresponding to the previously observed stimulus trajectory. Importantly, this “preplay” activity was not elicited when the stimulus was flashed at the end point of the moving dot sequence (Fig. 2). This same phenomenon has previously been directly observed in the firing activity of an ensemble of V1 neurons in rodents^52^, ^122^ and in macaque V4.^134^ Gavornik and Bear^52^ point out that the temporal specificity of these sequence representations are not predicted by Hebbian plasticity. Chong *et al*.^135^ measured V1 BOLD responses to an apparent motion illusion composed of spatially separated presentations of a rotating grating. V1 activity was found to contain an interpolated representation of an intermediate grating orientation along the illusory motion trajectory between the two grating presentation locations. Given that this intermediate grating was never presented to participants in the experiment, this result suggests that the brain reconstructs dynamic object features in V1, predicting current sensory input based on a representation of the stimulus trajectory.

Further evidence of neural prediction signals has been uncovered in research examining the substantial locomotor contributions to activity in rodent V1. These studies cleverly exploit a divergence between traditional models of sensory processing and PP by using self‐motion as a proxy for motor‐related sensory prediction.^41^ Although traditional feedforward models do not predict a difference in neuronal responses in early visual areas to visual flow depending on whether it is self‐generated or externally caused, PP suggests that error units will signal any deviation between sensory predictions and sensory input. Indeed, when mice run through a virtual environment, neural activity in V1 is significantly modulated by locomotor feedback carrying information about expected patterns of stimulation.^47^, ^48^, ^124^, ^136^ For example, the activity of anterior cingulate cortex (ACC) axons projecting to rodent V1 has been shown to convey predictions of upcoming grating stimuli^47^ or visual flow^124^ based on the mouse's movements. The V1 neurons targeted by these backward connections have been found to signal the mismatch between predicted and actual sensory input for their corresponding region of the visual field, characteristic of PP error units.^48^, ^98^ These predictions do not simply modulate visual responses but appear to drive the activity of V1 neurons even in the absence of bottom‐up input.^48^, ^136^ Moreover, these axonal influences are found to be experience dependent.^47^, ^123^, ^124^ For example, Leinweber *et al*.^124^ found that this predictive feedback to V1 came to reflect a new visuomotor coupling when a mouse was trained in an inverted virtual environment. Overall, these studies are not consistent with models characterizing primary visual cortex as a passive feedforward filter.^128^

At the same time, demonstrations of top‐down predictive influences on aspects of sensory processing do not necessarily provide definitive evidence for PP. For example, although Saleem *et al*.^136^ identified a small number of neurons sensitive to mismatches between input and expectations in V1, they found that the feedback modulation of the wider V1 population was better accounted for by a positive linear weighted sum of sensory input and predicted input than by prediction error. In addition, PP mismatch responses are typically considered to be the product of a simple subtraction (error = actual input – predicted input), but this scheme would appear to necessitate negative firing rates for omitted stimuli or for weaker than expected sensory inputs. In fact, several rodent studies have observed just such a response to an expected but omitted stimulus.^47^, ^48^, ^98^, ^123^ Keller and Mrsic‐Flogel^41^ accommodated the representation of signed prediction errors by proposing that PP is subserved by two classes of error unit: positive error units (excited by bottom‐up sensory signals and inhibited by top‐down prediction) and negative error units (with the opposite mapping). However, a recent study by Spratling^137^ showed that simulations using an alternative PP model,^2^ which uses division rather than subtraction to calculate error, better fit the neurophysiological data derived from these rodent studies than either Rao and Ballard's original model^18^ or Keller and Mrsic‐Flogel's revised model.^41^

### The development of prediction

According to PP, the range of interpretations of sensory stimulation entertained by the perceptual system is constrained by prior experience. Some priors can be bestowed through phylogenetic development and hardwired into neural structure,^138^ while others will emerge from persistent regularities in natural sensory experience and will likely be instantiated in the tuning properties of sensory cortex.^4^, ^19^, ^139^ For example, the prevalence of cardinal orientations in the sensory environment is reflected in the more narrow tuning and overrepresentation of neurons selective for cardinal orientations in early visual areas.^140^ Berkes *et al*.^141^ found that spontaneous activity in ferret V1 becomes increasingly consistent in response to natural scenes over the course of development, indicating that the visual system converges on a response shaped by prior sensory experience.

Nonetheless, such consistently reinforced priors appear malleable in the face of new experience,^6^, ^142^, ^143^ which suggests they may be represented both in the architecture of sensory cortex and in more dynamic, context‐sensitive top‐down influences. In this way, priors based on recent patterns of stimulation or current context can also influence sensory processing,^2^, ^144^, ^145^, ^146^, ^147^ even when regularities are embedded in complex naturalistic stimuli.^139^ For example, Li and Di Carlo^148^ designed a paradigm that exploited the fact that object representations in IT are robust to changes in viewing angle. As monkeys freely viewed a monitor, an image of an object was presented 3° displaced from their retinal position. When the monkey spontaneously saccaded to the image, the identity of the object was alternated during saccade execution. Subpopulations of neurons that initially exhibited a postsaccadic response preference for the first object identity, came to incorporate both object identities equally after repeated exposure, which is consistent with the idea of a flexible predictive model capable of updating stimulus expectations to reflect sensory regularities.

Several rodent studies have provided evidence that predictive activity emerges over the course of exposure to statistical regularities in visual stimuli^47^, ^52^, ^124^ (see also Ref. 88 for single unit recording in monkeys) and that this correlates with the magnitude of prediction error responses.^47^, ^123^ In line with PP's precision‐weighting mechanism, there is also evidence from auditory studies in humans that the inherent predictability of a stimulus stream moderates this process of prediction generation^144^, ^149^, ^150^ and subsequent ES.^151^, ^152^, ^153^ For example, Southwell and Chait^154^ found that the neural response to frequency outliers was enhanced when the deviant tones were presented in the context of a regular pattern compared with a random pattern even when the pattern was presented too rapidly to allow conscious detection of the regularity.

A particularly perplexing set of results in this literature comes from a series of studies reporting that the transition from random to regular sequences of auditory stimuli is accompanied by a sustained increase in neural activity measured with MEG, EEG, and fMRI,^144^, ^154^, ^155^ in stark contrast to ES studies showing dampened neural responses to predictable stimuli.^78^, ^81^, ^156^, ^157^ One interpretation of this result is that the enhanced activity reflects the high precision‐weighting afforded to low variance input.^155^ However, since precision‐weighting is applied to error units, this explanation of the sustained error signals rests upon the assumption that the increased precision more than compensates for the reduction in prediction error that would be expected over the course of exposure to a repeating, and therefore highly predictable, pattern of tones.

Finally, recent studies provide evidence of prediction error updating perceptual hypotheses to improve future predictions.^69^, ^158^ For example, Tang *et al*.^69^ applied a forward encoding model to EEG data in order to measure the orientation selectivity of visual signals in response to pairs of oriented Gabors. Unexpected orientations led to increased orientation selectivity soon after stimulus presentation and this effect reemerged at later time points, consistent with an updating of sensory expectations. Overall, this evidence is consistent with PP's contention that the brain constructs dynamic representations of regularities in the sensory stream and that these representations are intimately involved in subsequent sensory processing.^144^, ^154^, ^159^

### Hypothesis 2: Summary and conclusions

Humans are capable of rapidly extracting regularities from the sensory environment^144^, ^158^, ^160^, ^161^ and there is strong evidence that the resultant expectations influence sensory processing.^154^ It is a substantial achievement to begin to isolate this predictive activity in recordings of neural activity spanning human, monkey, and rodent research. Consistent with PP, neural activity believed to represent prediction appears to carry stimulus‐specific information, which is heavily experience dependent, and interacts with bottom‐up sensory input. However, there are many questions still to be answered. Although significant progress has been made,^4^, ^42^, ^162^ the neurophysiological mechanisms responsible for the extraction of regularities and the generation of experience‐based priors are not well understood. Although a range of candidate neural regions have been implicated in tracking a variety of sensory regularities and issuing predictions^29^, ^37^, ^40^, ^53^, ^55^, ^75^, ^120^, ^144^, ^158^, ^162^ (see Ref. 41 for a recent review), their precise relationships to the modulations observed in sensory processing are not yet established. Finally, it is not clear whether the formation of sensory predictions is a unitary neural process or an array of independent, task‐tailored mechanisms.^161^ Expectations can be formed in relation to stimulus timing, stimulus location, or stimulus content,^162^ and electrophysiological evidence suggests that these kinds of predictions are instantiated by distinct neuromodulatory mechanisms, in dissociable networks and at different latencies.^44^, ^162^, ^163^ Similarly, expectations can arise from arbitrary stimulus pairings,^90^ predictive cues, or higher order regularities.^66^ To what extent these forms of prediction rely on the same basic neural mechanisms remains to be determined.

## Hypothesis 3: Each level of the cortical hierarchy houses two functionally distinct neural subpopulations representing predictions and prediction errors

A central postulate of PP is that prediction error minimization is coordinated between two functionally distinct prediction and error unit subpopulations, which propagate signals across different frequency bands and cortical layers.^9^, ^40^, ^42^ However, it is only very recently that empirical research has directly addressed this hypothesis.

### Functionally distinct units

A corollary of PP's specification of two distinct subpopulations is that neurons encoding expectations should not encode prediction error and vice versa. Indeed, studies of primate and rodent neuroanatomy indicate that forward and backward projections originate from separate cell populations,^164^, ^165^ as PP requires. As we have seen, some fMRI studies have attempted to account for these subpopulations in their analyses (e.g., Refs. 76 and 87), but ultimately, BOLD responses will not adequately segregate their output as each voxel would contain an uncertain mixture of error and expectation units. More compelling evidence of functionally distinct subpopulations has been acquired through examination of single‐unit recordings. For example, Bell *et al*.^88^ found no correlation between the activity of a subset of neurons that encoded stimulus probability (i.e., prediction) and a subset that encoded prediction errors in macaque IT. Also in line with the predictions of PP, neurons that responded most strongly to faces showed the greatest difference in responses between expected and unexpected face trials, a characteristic of error units. Fiser *et al*.^47^ observed a similar result using calcium imaging to record neural activity in layer 2/3 of mouse V1 while the mice explored a virtual tunnel with oriented gratings (A or B) spaced out along the walls. They identified a group of V1 neurons, resembling expectation units, that exhibited orientation‐selective activity in anticipation of an upcoming grating over the course of exposure. Another group of neurons, resembling error units, exhibited stimulus‐evoked activity that was selective for a particular orientation and this selectivity was not substantially altered by experience. When an unexpected grating was shown in the final location, expectation neurons fired as if the predicted grating had been presented, while the activity of the orientation‐selective error neurons was driven by the actual stimulus. Strong feature‐selective predictive activity was associated with greater suppression of activity in error neurons with corresponding stimulus preferences in trials where the stimulus matched expectations. Conversely, when expectations were violated, stronger predictive activity preceded enhanced error unit responses. In line with the precision‐weighting mechanisms described by PP, Fiser *et al*.^47^ reported that the strength of predictive activity in V1 neurons and ACC axons projecting to V1 was greater as the mouse approached a position where the identity of the grating stimulus was reliably stable compared with a position where its identity varied. Finally, when the stimulus in the final position was omitted, a strong V1 response was observed and the response strength positively correlated with the strength of the preceding predictive activity. In fact, several studies of locomotor feedback in rodents have identified subpopulations of V1 neurons that signal the magnitude of the mismatch between expected patterns of visual flow and actual sensory input.^48^, ^123^, ^124^ Such activity profiles are consistent with PP and difficult to explain in terms of pure feedforward drive.

### Laminar segregation

The laminar segregation of bottom‐up and top‐down activity is an established feature of cortical anatomy.^164^ Forward signals are primarily transmitted from superficial to middle layers, while backward connections terminate in superficial and deep layers (Fig. 3A).^166^, ^167^, ^168^ The recent advent of high‐resolution neuroimaging has made it possible to exploit this laminar architecture to dissociate putative prediction and error signals in human subjects. Using 7T fMRI, Kok *et al*.^169^ found selective increases in activation of the deep layers of V1 induced by illusory figures associated with a Kanisza triangle, while dampened activity was observed in regions of superficial and middle layers of V1 associated with the encoding of the Pac‐Man inducers that resembled the laminar profile of genuine bottom‐up stimulation. This appears to accord well with PP: expectations are relayed from higher‐level areas to deep layers of V1 and the error units’ response to the predictable inducers is suppressed in the same layers as the response to the actual visual stimuli (Fig. 3C). However, it is not clear why the absence of bottom‐up sensory input at the location of the illusory contours did not itself evoke a prediction error response in the superficial and middle layers. In another high‐resolution fMRI study, Muckli *et al*.^132^ showed that only the superficial layers of V1 were significantly activated by a subregion of a visual scene that had been occluded (Fig. 6B). The fact that no subjects exhibited significant activity in the midlayers, where forward information peaked, suggests that this activity may represent descending predictions about the omitted scene. Interestingly, however, there was little evidence of a representation of the scene in deep layers where expectation units are thought to predominate. Although these studies demonstrate the promise of laminar fMRI as a novel means of investigating PP, neither can be said to offer concrete (dis)confirmatory evidence.

### Oscillations

In the primate visual system, signals transmitted forward through hierarchical levels are expressed in theta‐band (4–8 Hz) and gamma‐band (>30 Hz) activity, while backward signaling is carried out through beta‐band activity (12–30 Hz).^42^, ^170^ PP associates this oscillatory segregation between forward and backward processing^171^ with the distinct subpopulations housing error and expectation units, suggesting that oscillatory signatures of error and prediction message passing should be dissociable.^40^, ^42^, ^172^ Consistent with the proposed laminar profile of PP dynamics, evidence from monkey and human studies indicate that gamma‐band activity predominantly emanates from forward projections in superficial layers, while descending activity, carried by alpha/beta‐band activity, is strongest in layers 5/6.^40^, ^170^, ^173^, ^174^, ^175^ Studies that have sought to test PP's characterization of oscillatory message passing have found that when a stimulus is expected, beta power gradually builds in the lead up to stimulus onset, and gamma activity is reduced when those expectations are realized.^45^, ^176^, ^177^ Conversely, the violation of expectations is typically associated with increases in gamma power, while beta oscillations are initially reduced before resynchronizing.^172^ For example, Arnal *et al*.^84^ found that audio–visual mismatches evoked increased gamma activity in auditory cortex, which scaled with the strength of the predictive information. Fujioka *et al*.^178^ reported that beta‐band activity diminished after each beat of a rhythmic tone and reached a peak just before the next beat, suggesting it may be entrained to the tempo of the beat. When the tone was omitted, the beta‐band activity did not decrease, perhaps reflecting the recalibration of temporal inferences, and a sudden peak in gamma‐band activity was observed.

A number of other studies have provided evidence consistent with the idea that gamma‐ and beta‐band activity constitute prediction error and expectation signals, respectively. For instance, analyses of Granger causality by Richter *et al*.^179^ indicated that top‐down beta‐band activity targeting macaque V1 enhanced subsequent, spatially specific, stimulus‐driven ascending gamma‐band signals from V1 to V4 in a manner consistent with precision‐weighting mechanisms invoked by PP. Similarly, Sedley *et al*.^180^ recorded local field potentials using electrocorticography (ECoG) in three human subjects listening to auditory sequences with occasionally deviant fundamental frequencies. They demonstrated that human gamma‐band activity showed a positive correlation with an estimate of precision‐weighted prediction error derived from a Bayes‐optimal model, while beta‐band activity correlated positively with a model estimate of prediction updating. A recent study by Chao *et al*.^158^ isolated three components in macaque ECoG recordings of novelty responses in an auditory paradigm, whereby both established local and global regularities can be respected or violated (Fig. 4). They identified two gamma‐band components that closely matched model estimates for lower‐ and higher‐level prediction error signals, as well as a beta‐band component interpreted as a prediction update signal. When both local and global regularities were violated, the strength of the prediction update signal significantly affected the lower‐ and higher‐level prediction error signals on the subsequent trial. When just the global regularity was violated, prediction update signals only influenced higher‐level prediction error signals on the next trial. Chao *et al*. interpreted these findings as evidence that oscillations of distinct frequency channels transmit signals from expectation and error units across the cortical hierarchy. However, there are also some notable discrepancies between these studies. For instance, Sedley *et al*. reported a positive relationship between beta power and prediction updates, whereas Chao *et al*. found that prediction updates were associated with a reduction in beta power. Additionally, Sedley *et al*. associated prediction precision with alpha‐band activity, while Richter *et al*. linked enhanced precision to elevated beta‐band influences.

Other auditory studies have not observed these distinct oscillatory signatures or have reported conflicting findings. Using ECoG recordings, El Karoui *et al*.^181^ found that local deviants (i.e., a tone differing from the preceding tone in a sequence; see Fig. 4A) evoked high‐gamma responses in temporal cortex, consistent with previous studies, but global deviants elicited sustained decreases in beta activity. However, as the task in this study was to identify global deviants, it is difficult to ascertain how much of this effect is attributable to prediction and how much to changes in attention. Todorovic *et al*.^45^ found greater gamma power for unexpected tone repetitions and unexpected omissions, but unexpected repetitions also elicited greater power in low frequencies (5–9 Hz). In a subsequent study, Todorovic *et al*.^106^ found that beta activity decreased after unexpected tones, like Chao *et al*., but only in the absence of attention. If gamma‐band oscillations represent the output of error units, unpredictable deviants would be expected to provoke strong gamma‐band activity, while predictable deviants should elicit a more muted response. Although Durschmid *et al*.^182^ did find that high‐gamma (>60 Hz) responses differentiated between unpredictable and predictable deviant tones at frontal sites, unpredictable and predictable deviants elicited equivalent high‐gamma activity over temporal cortex.

### Hypothesis 3: Summary and conclusions

PP's specification of distinct neural subpopulations firmly divorces the theory from traditional models of sensory processing but also raises a critical methodological hurdle for those seeking to test its tenets. As highlighted above (Hypothesis 1), the question of distinct neural subpopulations presents a serious complication for the interpretation of global, noninvasive brain recording data and, therefore, in this section we have considered single‐unit recording studies that have attempted to dissociate these subpopulations. In fact, this issue also presents significant challenges for direct recording studies: if prediction and error units do exist, then definitively testing PP hinges on first ascertaining their prevalence in the sampled population. Acquiring recordings from distinct cortical layers, whether directly or indirectly, may be essential to overcoming many of these obstacles and adding to what is currently a limited evidence base supporting the existence of functionally distinct subpopulations (e.g., Refs. 47 and 98).

Although the assignment of forward and backward pathways to superficial and deep layers respectively is likely an oversimplification,^172^, ^183^ there is evidence that where these streams lie in adjacent layers, or even within the same layers, they remain segregated.^164^ There is also evidence of a segregation of prediction and error processing into oscillatory bands, but this finding is not universal and some of the discrepancies in the literature are difficult to reconcile.^42^ Moreover, it is not known precisely what kind of information is carried in these channels and there is little work describing their mutual influences.^158^ Furthermore, while gamma‐ and beta‐band activity have been proposed to support PP mechanisms, other frequency channels may also contribute to this activity.^172^, ^180^ Indeed, if PP is a canonical computation, *all* frequency channels would represent some form of inferential processing. Finally, it should be noted that while the presence of subpopulations of error and expectation units is a necessary characteristic of PP, the laminar and oscillatory asymmetries are also compatible with alternative functional architectures (e.g., Ref. 168) and even vary across some implementations of PP (see Ref. 103).

## Hypothesis 4: Prediction error minimization is achieved by an inferential hierarchy

As the sections above outline, many studies have identified expectation‐related modulations of neural activity that are consistent with PP. However, a central contention of PP models is that perceptual processing is coordinated by an inferential hierarchy.^17^ This hierarchy allows regularities at different spatiotemporal scales to exert an influence at an appropriate level of processing, funneling processing toward an internally consistent, globally coherent representation with each iteration. Top‐down influences are increasingly recognized as an integral component in early sensory processing,^41^, ^128^, ^184^, ^185^, ^186^, ^187^ but the contention that these hierarchical dynamics represent prediction error minimization has rarely been explicitly tested.

One avenue for investigating such a framework is to consider the relative latencies of expectation effects. PP suggests that RS occurs because a repeated stimulus becomes expected and, therefore, RS and ES are framed as arising from a common mechanism but one that is applied at distinct levels of the processing hierarchy.^27^ Although ES is characterized as a higher‐level effect, sensitive to complex regularities over extended periods of integration, RS is considered an automatic expression of low‐level prediction error minimization, sensitive to simple, transitional statistics.^55^, ^188^ Consistent with PP, numerous studies using MEG/EEG, fMRI, and ECoG show that while local deviants and simple regularities (e.g., repetitions) modulate early phases of neural responses, evidence of sensitivity to more complex sensory inference (e.g., global deviants and ES) emerges at later latencies.^46^, ^69^, ^81^, ^158^, ^181^, ^189^, ^190^, ^191^, ^192^, ^193^ Accordingly, early responses to local deviants are typically confined to primary sensory cortex, while violations of complex regularities evoke activity across distributed, higher‐level areas (e.g., Ref. 182; see Ref. 42 for a review). However, it is not clear to what extent these effects can be interpreted as confirmation of a hierarchical PP network.^42^ For example, there is substantial uncertainty about what exactly RS represents^27^, ^72^, ^194^ and PP and adaptation offer apparently indistinguishable accounts of expectation‐independent RS effects. Therefore, the observation that repetition‐ and expectation‐related suppression arise from lower and higher levels of the cortical hierarchy cannot be taken as definitive support for PP.

Although there is evidence that the predictability of a stimulus modulates activity in low‐level sensory areas^103^, ^128^ and alters the functional connectivity between neural regions associated with its processing,^75^, ^83^, ^155^, ^195^ efforts to trace the diffusion of these effects across the processing hierarchy have produced mixed results.^60^, ^61^, ^62^, ^67^, ^79^ For example, Utzerath *et al*.^67^ found evidence of RS, ES, and an interaction between expectation and repetition in area LOC. No evidence of RS, nor an interaction between repetition and expectation, was observed in V1, while ES was only detected in right V1. However, it is not clear that predictions about the kinds of complex object images used in these studies should be expected to suppress activity at the earliest stages of sensory processing (Fig. 6D). There is also a reasonable concern that stimuli labeled “unexpected” are often better described as “less expected.” In the Utzerath *et al*. paradigm, the stimulus pool was composed of just four stimuli and the “unexpected” transitions occurred in 25% of trials. Given the limited range of outcomes, it may be that the stimulus probabilities had a marginal effect on processing and the associated early visual responses.

Another intriguing question raised by PP is how does a higher‐level area, with its own stimulus preferences, communicate predictions about activity at a preceding level of the hierarchy with distinct response properties? Recent invasive neural recordings have attempted to capture the unfolding of these hierarchical interactions over time. Schwiedrzik and Freiwald^92^ sought to identify signatures of this process across three levels of the macaque face‐patch system, having trained monkeys to associate nine pairs of face stimuli with differing facial identities and head orientations. These three areas are distinguished by their increasingly abstract stimulus preferences: ML, whose neurons exhibit viewpoint specificity; AL, which is characterized by mirror‐symmetric response preferences (i.e., profile‐selective); and AM, which is tuned to view‐independent stimulus identity.^73^ Since minimizing the error response to a view violation may require only the revision of sensory expectations at the lowest level of the hierarchy, these errors might be expected to be resolved more quickly than those requiring updates to higher‐level predictions. Indeed, the results showed that ML responses to simple violations of head orientation diminished more quickly than more complex stimulus violations involving discrepant facial identities (Fig. 5). In addition, the authors found that mirror‐symmetric view violations produced smaller responses in ML than nonmirror‐symmetric violations in the late phase of the response. Together, these results could be interpreted as evidence that early responses reflect the local tuning properties of error units, while activity in the later phase of a response represents the involvement of higher‐level areas (AL) in the resolution of more complex prediction errors. However, an important detail of this study is that the deviant stimulus pairings were not coded according to the departure of the second face from the expected second face but as the departure of the first face from the normal predictor of the second face. Because the deviant predictor stimuli would, on a subset of trials, also predict a different view to that which appeared, many of the trials coded as “identity violations,” could be classed as “identity and view violations,” which may offer an alternative explanation for the similarity between the average response to these trial types. Moreover, some of the trials in the “view violation” condition required predictors that were not among the pool of trained predictor stimuli and may therefore have failed to evoke any specific expectations.

In another study, Issa *et al*.^196^ pointed out that PP also diverges from other accounts in predicting enhanced activity over time from low‐level error units that prefer the presented stimulus features, when the stimulus conflicts with sensory predictions from higher levels. To test this, they simultaneously recorded neuronal activity from three hierarchical levels of the macaque face processing system while presenting typical and atypical configurations of macaque facial features. The results showed that neurons in lower‐level face‐selective subregions of IT increasingly responded to atypical configurations of facial features in the late phase of the response, while the higher‐level area maintained a preference for typical facial configurations throughout. Furthermore, the late‐phase response in the lowest level was better predicted by the early response in higher levels than the lowest level itself, consistent with the idea that updated sensory expectations are distributed from higher levels to resolve lower‐level error responses. Issa *et al*. went on to show that these hierarchical neural dynamics could not be captured by feedforward models incorporating lateral inhibition, normalization, or additional excitatory feedback representing Bayesian inference, but were consistent with a PP model describing the lower‐level late‐phase activity as a prediction error response.

According to PP, the conditional probabilities afforded by higher‐level representations may be exploited to constrain lower‐level sensory representations, promoting contextual congruence across levels of the hierarchy.^4^, ^132^ Indeed, there is evidence that the neural representation of simple stimulus features is influenced by higher‐level object representations^115^, ^129^ and object representations are modulated by scene context.^197^ For example, Harrison *et al*.^198^ found that the global coherent motion of a random‐dot kinematogram produced diminished responses in V1. Importantly, the constituent dots were deliberately spaced to extend beyond the classical receptive field of a V1 neuron, thus excluding lateral interactions as a potential explanation of this contextual suppression. Similar to the paradigm used by Issa *et al*., Hsieh *et al*.^199^ compared BOLD responses in early visual cortex with Mooney images (Fig. 6A) before and after human subjects recognized the stimulus as a meaningful image. They reported that when subjects could interpret what the two‐tone image represented, the response to this stimulus more closely resembled the response to a full grayscale version of the same image than the response to the identical two‐tone image before it was interpretable.

Similarly, others have found that the grouping of moving stimulus elements into a perceptual object is associated with increased activity in area LOC and suppressed activity in lower‐level areas hMT and V1 (Fig. 6C).^114^, ^115^ This observation accords with a predictive model in LOC transmitting its perceptual hypothesis to lower‐level areas to quell prediction error. Interestingly, these studies demonstrate prediction effects that complement those observed by Utzerath *et al*. in the same brain regions (see above). One explanation for a more modest effect of ES in V1 observed by Utzerath *et al*. compared with the robust effects reported by Murray *et al*.^115^ and Fang and He^114^ is that the conditions in these studies differed in terms of the presence or absence of a high‐level perceptual hypothesis rather than in terms of a more differential effect of expectation (75% likely versus 25% likely). Disrupting activity in V1/V2 and area LOC using transcranial magnetic stimulation (TMS), Wokke *et al*.^187^ found that the critical window for disrupting a Kanizsa illusion was earlier in LOC than V1/V2, suggesting early influences from backward connections play an essential role. Similarly, V1 activity representing an apparent motion illusion (e.g., Ref. 125) is influenced by early feedback from MT,^200^ and the detection advantage associated with targets predicted by the apparent motion trace is eliminated by applying TMS to MT before target onset.^186^

### Hypothesis 4: Summary and conclusions

Many studies that identify expectational modulations of neural activity struggle to demonstrate systematic effects across contiguous levels of the hierarchy (e.g., Ref. 78). However, as mentioned above, this may be partly due to the fact that it is highly challenging to establish precisely what, if any, predictions may be applied at a given processing level. For example, complex stimuli like faces may not yield any specific predictions at the level of detail pertaining to the simple stimulus preferences of V1 receptive fields.^67^, ^79^ Indeed, most of the studies that report PP‐like dynamics in V1 use simple stimuli like gratings or flashes.^51^, ^78^, ^107^, ^135^, ^201^ PP's stance on this problem is somewhat unclear. PP does not predict that the evidence for perceptual inference should be modular, siloed in an area of the brain specialized for the particular stimulus in question. However, more work is needed to understand how sensory predictions might be conveyed across processing levels^41^ and to demarcate the extent to which predictive activity should be detectable as one moves away from those specialized processing hubs.^202^

Overall, there is good evidence that the sensory brain is hierarchically organized and that it exploits descending predictive activity to render sensory signals into meaningful constructs. However, the evidence for a PP inferential hierarchy specifically is scarcer. Although this may reflect on the veracity of the theory, it may also be attributable to the great difficulty such a task poses.^161^ It is not sufficient to demonstrate that descending connections to early areas introduce contextual modulations that are essential for perception because this is accommodated by traditional models of visual processing (e.g., Ref. 131). What is required are further demonstrations of PP dynamics unfolding across simultaneous recordings from multiple cortical areas, such that the hierarchical interplay of prediction error and prediction can be verified (e.g., Ref. 196). Efforts to increasingly approximate this standard of evidence may also shed light on the important question of how different neural areas communicate prediction in meaningful ways.^203^

## Discussion

Traditional theories of perceptual processing have failed to adequately explain why sensory cortices are infused with masses of descending connections.^10^, ^32^, ^204^ For example, backward projections from V2 to V1 are 10 times more numerous than forward projections from the lateral geniculate nucleus to V1^205^ and the processing of ascending sensory signals is estimated to account for only 1–2% of the brain's energy consumption.^117^ Conversely, PP provides a satisfying explanation of these copious backward connections, impugning their consignment to mere recurrent modulations in feedforward schema.^183^, ^206^ The explanatory power of this framework, exemplified in simulations of PP that readily account for phenomena ranging from V1 neuron response properties^207^ to bistable perception^29^ to perceptual illusions,^30^, ^31^ has led some to argue that everything the brain does can ultimately be explained in terms of prediction error minimization.^9^, ^17^, ^32^ Such a bold contention has inevitably drawn skepticism, much of which has centered on the theory's empirical foundation.

Without question, PP can comfortably accommodate many neurophysiological observations in research examining the role of expectation in perception. But, in reality, few studies have set out to explicitly test hypotheses that are unique to PP and many empirical observations interpreted as supporting PP are derived from paradigms ill‐equipped to discriminate among competing models. A key goal of this review was to highlight a number of recent neurophysiological investigations that have leveraged novel paradigms and technological advances in order to put PP to the test. Although on the whole it must be concluded that the empirical support for each of PP's key hypotheses is mixed, there is much work yet to be done and it is striking that clear‐cut counterevidence has yet to emerge.

In fact, a common criticism of PP is that it is very difficult to falsify.^34^ PP's specification at the level of an algorithm can be seen as a virtue, providing a framework for the integration of explanations at multiple levels from synapse to processing hierarchy,^7^, ^208^ but it can also pose a challenge as researchers seeking to test the theory's validity confront the often murky translation from algorithm to biophysical implementation. Indeed, critiques of the standard implementation of PP,^18^ focusing on the model's biological plausibility (e.g., neurons with both positive and negative firing rates), have spawned numerous alternative versions with revised circuitries and diverging predictions.^2^, ^41^ Confusion can sometimes arise where it is not clear which of these various incarnations are being tested in a particular study. In addition, the flexibility conferred on PP models by hypothesizing functionally distinct neural subpopulations and layering a precision‐weighting mechanism on their responses at every level of the hierarchy renders the theory capable of accommodating apparently contradictory results.^34^

A necessary next step, therefore, is to provide definitive evidence of the existence of expectation and error units in neural processing. Although this represents a considerable challenge using conventional paradigms and neurophysiological techniques, methodological advances in high‐resolution fMRI, optogenetics, calcium imaging, and serial single‐unit recordings at multiple levels of the processing hierarchy are providing powerful new opportunities to trace neural markers of hierarchical PP dynamics.^41^, ^128^, ^158^, ^196^, ^209^, ^210^ Pairing these increasingly sophisticated neural assays with anatomical models, computational modeling, and simulations^137^, ^211^ will enable researchers to derive fine‐grained *a priori* hypotheses and compare model evidence for variant architectures and also for near‐variant ones that share much with the core PP picture but differ in their conceptions of the encoding, flow, or use of prediction errors (e.g., Refs. 2, 10 and 11; see Ref. 137). As the theory is refined by these more delicate neurophysiological tests we hope to see a bridging of the gaps between parallel literatures that currently exist somewhat in isolation^212^ (e.g., perceptual decision making).

Although the debate about PP's empirical grounding is currently unsettled, the theory can nevertheless be regarded as a milestone in cognitive neuroscience, spurring efforts to recognize the importance of backward connections in the architecture of the neocortex and the role of prediction in sensory processing.^206^, ^213^ Does PP reflect a questionable commitment to bringing multiple phenomena under a single unifying umbrella—one that may not be able to do full justice to any of them? Or can a mature PP capture the full sweep of behavioral effects and experimental data, revealing them as flowing from a core rationale expressed using a handful of repeated processing motifs? Whatever the outcome, this is a lively and ever‐expanding literature that allows us to revisit many long‐standing assumptions regarding neural function, the nature of mind, and the origins of human behavior.

## Competing interests

The authors declare no competing interests.
